# Modeling the Impact of Retention Interventions on Mother-to-Child Transmission of HIV: Results From INSPIRE Studies in Malawi, Nigeria, and Zimbabwe

**DOI:** 10.1097/QAI.0000000000001364

**Published:** 2017-05-15

**Authors:** Elizabeth McCarthy, Jessica Joseph, Geoff Foster, Alexio-Zambezio Mangwiro, Victor Mwapasa, Bolanle Oyeledun, Sam Phiri, Nadia A. Sam-Agudu, Shaffiq Essajee

**Affiliations:** * Applied Analytics Team, Clinton Health Access Initiative, Lusaka, Zambia;; † Applied Analytics Team, Clinton Health Access Initiative, Boston, MA;; ‡ Ministry of Health and Child Care, Mutare, Zimbabwe;; § Family AIDS Caring Trust, Mutare, Zimbabwe;; ‖ Clinton Health Access Initiative, Harare, Zimbabwe;; ¶ Department of Public Health, College of Medicine, University of Malawi, Blantyre, Malawi;; # Centre for Integrated Health Programs, Abuja, Nigeria;; ** Lighthouse Trust, Lilongwe, Malawi;; †† Department of Medicine, University of North Carolina School of Medicine, Chapel Hill, NC;; ‡‡ Department of Public Health, School of Public Health and Family Medicine, College of Medicine, University of Malawi, Lilongwe, Malawi;; §§ International Research Center of Excellence, Institute of Human Virology Nigeria, Abuja, Nigeria;; ‖‖ Division of Epidemiology and Prevention, Institute of Human Virology, University of Maryland School of Medicine, Baltimore, MD; and; ¶¶ World Health Organization, Geneva, Switzerland.

**Keywords:** implementation science, retention, HIV, Africa, PMTCT, mathematical modeling

## Abstract

**Background:**

Six implementation research studies in Malawi, Nigeria, and Zimbabwe tested approaches for improving retention in care among women living with HIV. We simulated the impact of their interventions on the probability of HIV transmission during pregnancy and breastfeeding.

**Methods:**

A computer-based state-transition model was developed to estimate the impact of the retention interventions. Patient-level data from the 6 studies were aggregated and analyzed, and weighted averages of mother-to-child transmission (MTCT) of HIV probabilities were presented. The average MTCT probability of the more successful interventions was applied to national estimates to calculate potential infections averted if these interventions were taken to scale.

**Results:**

Among the total cohort of 5742 HIV-positive women, almost 80% of all infant infections are attributed to the roughly 20% of HIV-positive pregnant and breastfeeding women not retained on antiretroviral therapy. Higher retention in the arms receiving interventions resulted in an overall lower estimated MTCT probability of 9.9% compared with 12.3% in the control arms. In the 2 studies that showed a statistically significant effect, Prevention of MTCT Uptake and Retention (PURE) and Mother Mentor (MoMent), the difference in transmission rates between intervention and control arms was 4.1% and 7.3%, respectively. Scaling up retention interventions nationally in the 3 countries could avert an average of almost 3000 infant infections annually.

**Conclusions:**

Linking HIV-positive pregnant women to antiretroviral therapy and retaining them is essential for addressing the remaining gaps and challenges in HIV/AIDS care and the elimination of MTCT. At national level, even modest improvements in retention translates into large numbers of infant infections averted.

## Abstract 2

Supplemental Digital Content is Available in the Text.

## INTRODUCTION

The INSPIRE (**I**ntegrating and **S**caling up **P**MTCT through **I**mplementation **RE**search) initiative comprised 6 implementation research studies—2 each in Malawi, Nigeria, and Zimbabwe.^1^ Each study tested its own innovative approach to improving retention in HIV care and treatment of HIV-positive mothers and their children in its prevention of mother-to-child transmission of HIV (PMTCT) program. Clinical trials and programs in developed countries have shown that it is possible to significantly reduce mother-to-child transmission (MTCT) of HIV, putting virtual elimination of MTCT within reach. These data inspired the “Global Plan,” an effort across 21 prioritized countries in sub-Saharan Africa and India to achieve a MTCT rate of 5% or less among breastfeeding populations and 2% or less among nonbreastfeeding populations by 2015.^2^ Significant progress toward this goal has been made, with a reduction from 28% in 2009 to 14% in 2014.^3^ However, the operational realities of implementing antiretroviral therapy (ART) programs and persistent retention challenges have attenuated the potential impact of expanded treatment access for pregnant and breastfeeding women and have kept the goal of virtual elimination just out of reach for many countries. By testing multiple innovations targeting improvements in service delivery and retention for HIV-positive women in 6 different real-world settings, the INSPIRE studies aimed to provide evidence to inform program design that will bring countries close to the goal of achieving virtual elimination of MTCT of HIV.

The primary outcome for the INSPIRE studies was retention in care among pregnant women and mothers. Accurate estimates of the impact of these efforts on transmission, measured by the number of newly HIV-positive infants, are critical for estimating the need for pediatric antiretroviral drugs (ARVs) and other resources such as the number of facilities and trained staff. Measures of success in preventing MTCT are also essential for policy makers and health care providers to assess the impact of their efforts and accordingly continue or modify their approach to PMTCT service delivery. The benefits of retention on ART are well recognized as beneficial for HIV-positive women in terms of their own health. The impact of improved maternal ART retention on ART for their infants is harder to measure; HIV testing rates of HIV-exposed infants remains low and often disproportionally, misses the children of women who have stopped coming to the facility and are more likely to no longer be on ART.

We developed the PMTCT and Pediatric Impact and Cost Model in 2010 to inform national health decision making regarding the elimination of MTCT and ART scale-up for pregnant women and children, with a focus on country-level adoption of the World Health Organization HIV treatment guidelines as they were released and maximizing the impact with available resources, particularly drugs, diagnostics, and health workers.^4–7^ A modified version of this model was developed to estimate the probability of HIV infections transmitted from mother to child within the context of the INSPIRE interventions in Malawi, Nigeria, and Zimbabwe. The modeled estimates of HIV transmission allow us to generalize the effect of the interventions in the INSPIRE studies beyond their current time frames and beyond the immediate populations of the 6 individual studies to better understand the potential long-term impact of investments in interventions to improve retention of HIV-positive pregnant and breastfeeding women and their infants. Understanding what is possible within the 6 studies allows for the reflection on how many infant HIV infections could be averted at a broader level if interventions to improve retention were taken to national scale.

## METHODS

We examined the relationship between MTCT and timing of maternal ART initiation and retention using a computer-based state-transition model using Markov cohort simulation to estimate MTCT probability among the cohort of women enrolled in the 6 INSPIRE studies.^8^ The study names, setting, intervention, design, and cohort size are described in Table 1.^9–14^

**TABLE 1.**
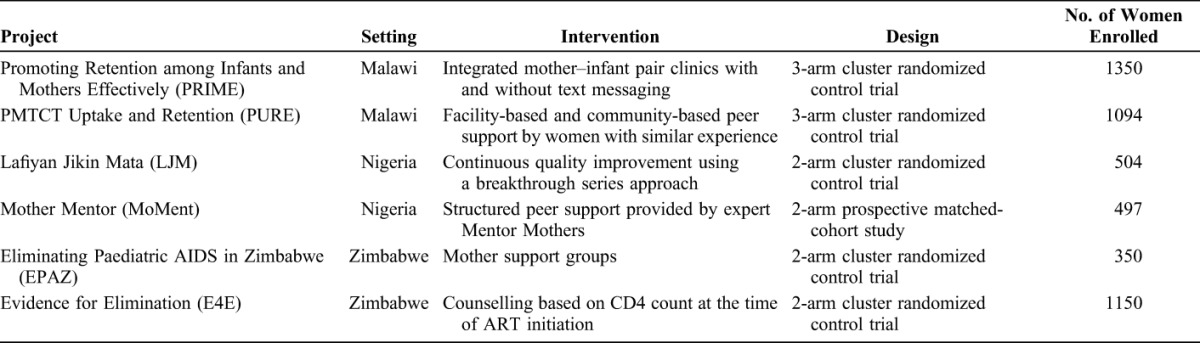
Description of INSPIRE Projects

### Data Collection

Aggregated patient data from the 6 INSPIRE studies were merged. Because outcomes and data collection processes of each study varied, some assumptions were necessary to integrate the data and produce comparable MTCT estimates for each study and overall for the 6 studies. For all INSPIRE studies except PMTCT Uptake and Retention (PURE) Malawi, we used national averages of monthly breastfeeding relevant to the study location. On average, 84% of women were assumed to be breastfeeding until 12 months postpartum in Nigeria and Zimbabwe, and 97% in Malawi.^15–17^ The PURE Malawi study estimated 12-month breastfeeding at 33%. Although 33% is much lower than the 97% national estimate from the 2014 Malawi Indicator Cluster Survey (MICS), we used the PURE study breastfeeding data for the PURE analysis because it was available for this study. Although Promoting Retention among Infants and Mothers Effectively (PRIME) study also took place in Malawi, we decided to use the MICS data rather than use PURE estimates for PRIME, given that MICS should be the most nationally representative in the absence of study data.

For studies without complete data for all women through 12 months postpartum, we used the data that were available for each month of follow-up and assumed the same pattern of retention for subsequent months as was observed in the last month of available data. This could have underestimated the number of infections from that study population if retention rates in reality continued to decline after data were collected.

For example, Lafiyan Jikin Mata Nigeria observed women for 6 months postpartum rather than 12 months.

Two of the studies (PURE and PRIME Malawi) had 3 arms, and the other 4 were 2-arm studies. To balance the comparison between intervention and control groups overall, we counted the control arms twice for the 3-arm PURE and PRIME studies, resulting in a total of 8 control and 8 intervention arms.

One of the important underlying assumptions of the model is that MTCT probability does not depend on prior obstetric or HIV treatment history.^8^ For example, the model did not account for prior pregnancies, age, date of enrollment, or maternal history with ART. We analyzed the population of HIV-positive women in each arm as a single cohort passing through the states at the same time. The ART retention states were assumed to be mutually exclusive and collectively exhaustive, meaning that the women must be in a retention state and could only be in 1 state at a time. Each state was homogeneous, meaning that every individual in that state had the same probability of transmission as the other. The women's characteristics themselves (ie, age, CD4 count, viral load, years of HIV infection, or prior pregnancies) were not assumed to determine the transmission probability; rather, they were assumed to have the same probability of transmitting HIV to their infant based on their ART and breastfeeding status.

### Transmission Probability Assumptions

In the absence of population-based epidemiological surveys, the transmission probability estimates of the model rely on clinical trials, mostly from sub-Saharan Africa, which were consolidated in 2012.^18^ Since that time, more recent evidence from published clinical trials has become available that builds on the previous synthesis.^19–21^ Average MTCT model assumptions were given a proportionate 30% upper and lower range to help account for uncertainty (Table 2).

**TABLE 2.**
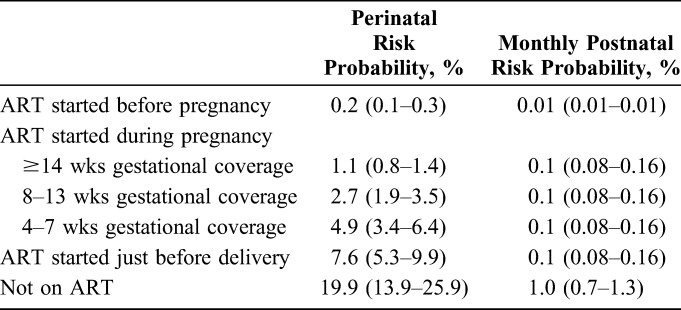
Summary of Transmission Probability Assumptions by Maternal ART Coverage During Perinatal and Postnatal Periods Inputted Into the Model

### Retention as a Proxy for ART Adherence

Each INSPIRE study used a different definition of PMTCT retention in care for pregnant and breastfeeding women and their HIV-exposed infants, related to the context in which the studies were implemented and the interventions that were tested (see elsewhere in supplement for additional detail). We assumed that if a woman was retained, then she was receiving ART and taking the drugs as prescribed. Our focus is on consistently taking ARVs in contrast to a definition of retention that could mean that a woman is attending an appointment as scheduled, picking up her ARVs on time, or attending a community support group. The focus on taking ARVs is critical given that this assumption forms the basis of the modeled transmission probabilities.

### Model Structure and Simulation

The model simulates a closed cohort of HIV-positive women during the pregnancy and delivery period and for 12 months after delivery, capturing MTCT risk over time (Supplemental Digital Content 1, http://links.lww.com/QAI/A993). We modeled the intensity (duration of protection during pregnancy and breastfeeding) of a therapeutic strategy (maternal ART) to prevent disease transmission, under the assumption that a more intense strategy (starting ART early and continuing throughout the entire breastfeeding period) would reduce the risk of the event of interest (HIV transmission to the infant). Risk probabilities were assigned to each state, whereby the number of new infant HIV infections depends on whether the mother is on ART and the duration of ART protection during pregnancy and delivery.

HIV-exposed infants, as a result of their risk of acquiring HIV via MTCT during pregnancy, delivery, and breastfeeding, comprised the cohort of interest. The model was used to predict the longer-term impact of retention strategies introduced in each INSPIRE intervention arm. The model tracked fetuses/infants as they started out HIV-negative, were exposed to HIV while in utero, during delivery, and during breastfeeding, accounting for different probabilities of HIV transmission depending on maternal use of ART and length of breastfeeding. The time horizon modeled included the gestational period and 12 months after delivery.

During pregnancy and delivery, women within the study control and intervention cohorts fell into 1 of 2 states: retained or not retained on ART. Here, ART referred to a triple combination of ARVs and did not include other interventions such as providing a single dose of maternal nevirapine at birth or providing maternal azidothymidine (AZT) during pregnancy. Women retained on ART were further differentiated into those who initiated ART *before* the current pregnancy (eg, were already on lifelong treatment) or initiated ART for the first time *during* the current pregnancy.

Among those who initiated ART during the current pregnancy, there were 4 states depending on the duration of coverage during pregnancy: those who were on ART for 14 weeks or more before delivery, between 8 and 13 weeks before delivery, between 4 and 7 weeks before delivery, and <4 weeks before delivery. The not retained population included women who were on ART during pregnancy but stopped ART before delivery and those who never received any ART during this current pregnancy. The latter category included women who continued to not receive ART during breastfeeding and those who would later start ART.

The categories of women were static during the pregnancy and delivery period. They become dynamic during the postnatal period, when in each month women/infants within the cohort had the possibility of transitioning from breastfeeding to not breastfeeding. Exact dates of stopping breastfeeding were not used. Rather, if a child was breastfeeding at any time in a month, he or she was counted as breastfeeding that month. If they stopped breastfeeding in a particular month, they were considered as not breastfeeding starting from the first full month when no breastfeeding occurred. Infants no longer breastfeeding were considered not at the risk of HIV transmission and were therefore excluded from the population at risk after breastfeeding cessation. Breastfeeding women could transition from being retained on ART to not retained. Women who were not retained on ART during pregnancy could transition to being retained during breastfeeding.

Postnatal transmission was calculated using a monthly probability of transmission based on retention on postnatal maternal ART during the breastfeeding period, plus the duration of breastfeeding. As with all estimates of disease incidence, only those exposed were included in the population at risk. In the case of MTCT, all HIV-exposed infants are at the risk of acquiring HIV while in utero and during delivery. Only breastfeeding infants not yet infected are at the risk of acquiring HIV after delivery. At 1 month postpartum, all children infected during pregnancy/delivery were excluded from the population at risk, as were those who were not breastfeeding. The at-risk population was adjusted each subsequent month to remove infants infected the previous month and those who stopped breastfeeding, continuing until the end of the observation period (12 months) or the end of the risk period (the end of breastfeeding).

### Analysis

We explored the probability of HIV transmission in the prenatal period compared with the breastfeeding period and compared those retained with those not retained. We assessed transmission in the integrated control group from the 6 studies compared with the integrated intervention group from the 6 studies. Given the variation in population size of each study, we weighted the integrated data results of HIV transmission probability based on the sample of enrolled mothers in each study. All analyses were performed with Microsoft Excel, Redmond, Washington.

## RESULTS

### ART Profile of Study Participants

Nearly 3000 women were included in both the merged 6-study control cohort (2785) and intervention cohort (2957) illustrated in Table 3. A relatively large proportion of HIV-positive pregnant women in each group (26.8% and 19.3% in control and intervention groups, respectively) were not retained on ART at the time of delivery. A proportion (17.7% in the control group and 10.8% in the intervention group) had actually initiated ART during the current pregnancy but stopped taking ARVs before delivery. Some (4.5% in each group) had started ART before this pregnancy and stopped before delivery, and some (4.6% and 4.1% in control and intervention groups, respectively) were not on ART at all during the pregnancy.

**TABLE 3.**
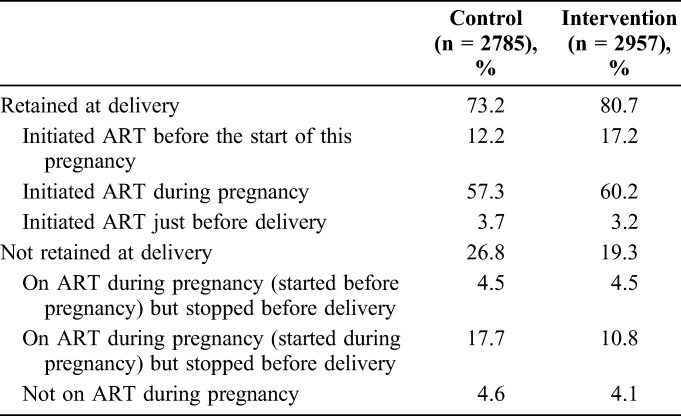
Characteristics of Timing of ART Initiation and Retention Among HIV-Positive Women in the INSPIRE Studies' Control and Intervention Groups

Of women retained on ART at delivery, the greatest proportion (57.3% in the control group and 60.2% in the intervention group) initiated ART during that particular pregnancy (although this would also be influenced by study-specific inclusion criteria). A smaller proportion (12.2% and 17.2%, respectively) were already on ART before the start of the pregnancy. Overall, only 3.7% and 3.2%, respectively, started in the 4 weeks just before delivery. On the whole, the profile of maternal ART initiation and stopping between control and intervention groups is very similar, with roughly 7% higher retention in the intervention group (Table 3).

### Proportion of Infant HIV Infections According to Retention Profile

On average, close to 80% of all infections are attributed to the roughly 20% not retained. Across both cohorts, overall between 77.3% and 80.6% of infant HIV infections occurred among women not retained on ART (Table 4). The majority of women were retained and make up only about one-fifth of the overall infections.

**TABLE 4.**
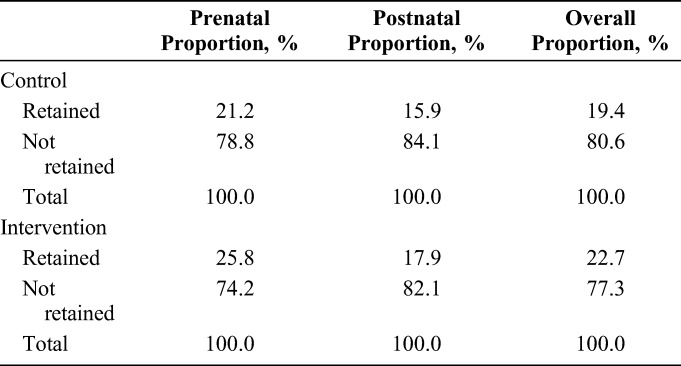
Proportion of Overall HIV Infections Occurring Within Each Retention Category and Each Risk Period by Control and Intervention Groups

### Probability of MTCT

In this first analysis, the difference in MTCT rates are estimated for intervention and control arms of all INSPIRE studies together even if individual studies did not report an impact on retention in care. The range around the MTCT estimate at each time point is relatively wide and overlapping, yet the estimates suggest that overall HIV transmission was roughly 2.4% lower in the intervention group at 9.9% (range, 7.0%–12.8%) compared with the control group at 12.3% (range, 8.6%–15.9%), as shown in Table 5. Both control and intervention groups had slightly higher transmission during pregnancy compared with the 12-month breastfeeding period. A stark difference in rates between those retained and not retained is visible, linking directly to the rates of transmission from the literature used in our model assumptions. The variability in the retained figures relate mostly to the timing of ART initiation (before this pregnancy, early in this pregnancy, or just before delivery), with the longer duration of ART coverage resulting in lower transmission.

**TABLE 5.**
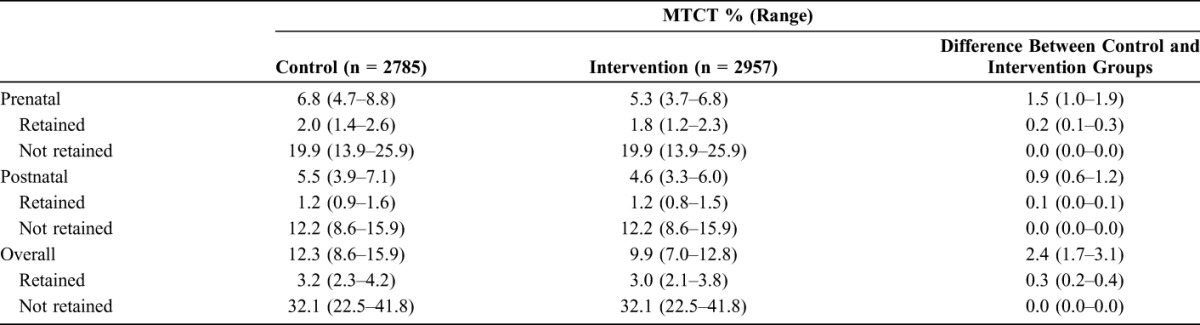
Modeled Probability of MTCT Among HIV-Positive Women in the INSPIRE Studies' Control and Intervention Groups

In a second analysis restricted to the 2 studies that showed significant improvements in retention associated with their interventions, PURE and Mother Mentor (MoMent), the average difference in MTCT rates between the intervention and control arms was 5.7% (range, 4.1%–7.3%).

### National-Level Impact of Improved Retention on ART During Pregnancy and Breastfeeding

In an analysis of national-level impact, we used the average difference in modeled retention rate (5.7%) from 2 studies (PURE and MoMent). We applied this average difference to 2015 national estimates of the number of children newly infected with HIV in the 3 INSPIRE countries.^22^ We found that almost 3000 new pediatric infections could be averted in 1 year across Nigeria, Malawi, and Zimbabwe if successful retention interventions were added to existing efforts in each country (Supplemental Digital Content 1, http://links.lww.com/QAI/A993).

## DISCUSSION

Among the total cohort, the majority of new pediatric infections were attributed to the approximately 20% of HIV-positive pregnant and breastfeeding women not retained on ART. Higher retention in all the intervention arms together resulted in a 2.4% difference in overall estimated MTCT probability compared with the control. This is probably an underestimate of potential benefit as the intervention cohort included mothers and infants of studies where there was no effect of the interventions tested by the individual studies. In the 2 studies, however, that showed a statistically significant effect, PURE and MoMent, the difference in transmission was marked with an average MTCT reduction of 5.7%. Scaling up these successful interventions nationally in the 3 countries could avert a large number of infant infections annually.

Models provide predictions, the accuracy of which depend on the underlying assumptions and how close they are to the real world.^23^ Here, we are modeling changes in rates of HIV transmission among children as a result of scaling up programmatic interventions to improve retention among pregnant women and mothers. Predictions that go beyond the measured study outcomes may assist with decision making about further investment in the interventions that were tested. However, it is critical that the model's predictions be qualified and explained and thus interpreted with the understanding that model results are approximations.

The INSPIRE studies implemented interventions to increase retention in care for improved maternal and infant health. Study populations included women living with HIV who were either initiated on ART or already on ART and those who stopped taking ART during pregnancy and/or breastfeeding. Our modeling confirms that in 3 countries, significant successes in terms of disease transmission can be realized when HIV-positive women receive ART. The studies all relied on existing health infrastructure and systems, and they provide a realistic picture of what can be achieved when retention investments complement existing efforts and the associated challenges. This is consistent with results from other countries in sub-Saharan Africa.^24,25^ Gains from improving retention among the nonretained may not be as striking as the effects of ARVs among retained populations, yet the focus now needs to shift to addressing the needs of that 20% of the population, contributing a disproportionate 80% of the infections.

The impact of retention interventions goes beyond the disease transmission that we have modeled here and extends to other maternal and child health outcomes. ART for HIV-positive women maintains better health, allows women to work and pursue their own ambitions, and is valuable to their lives apart from the benefit for their children. For children, beyond the HIV diagnosis, HIV-free survival has been shown to increase among those born to HIV-positive mothers the longer the period that women were on ART during pregnancy.^26^ Some of the interventions that reported no gain in terms of retention had effects on other outcomes such as early infant diagnosis rates and are likely to have an impact on pediatric ART initiation and longer-term morbidity and mortality outcomes.

The main limitations of this analysis are the underlying uncertainty around the epidemiological assumptions and the uncertainty around the inputs. There is thus uncertainty around the estimates of MTCT probability reflected as a wide range. In addition to these inherent limitations of the approach to modeling HIV disease transmission, we have introduced additional variability using integrated data from 6 distinct studies. Each study evaluated the impact of different interventions and had its own sample size, outcomes, and data collection methods. Assumptions were needed to standardize and pool individual study results for the purpose of analyses. To have a meaningful comparison, we doubled the numbers coming from control cohorts of the 3-arm PURE and PRIME Malawi studies, which, for this analysis, could overrepresent the standard of care in Malawi compared with Nigeria and Zimbabwe. In the absence of data on breastfeeding practices among women included in the studies for all except the PURE study, we assumed patterns similar to the national average, which may have overestimated or underestimated the percentage of women breastfeeding each month. This may be important because incident infections during breastfeeding are known to significantly contribute to MTCT of HIV. Given the challenge of collecting accurate data on women who seroconvert during pregnancy or breastfeeding, we have not adjusted for incident infections in this analysis.

At the national level, even modest benefits in retention translates into large numbers of infant infections being averted. Investment in retention interventions during the critical phase of pregnancy and breastfeeding is critical if the protective benefits of ART on HIV transmission and survival are to be realized. Drugs available but not taken do not prevent infections or improve health outcomes. The hard work of linking HIV-positive pregnant women to ART and retaining them must be a priority if national and global goals of elimination of MTCT and maternal and child survival are to be met.

## Supplementary Material

SUPPLEMENTARY MATERIAL
