# Supplementary figures and images for: Modeling the Impact of Retention Interventions on Mother-to-Child Transmission of HIV: Results From INSPIRE Studies in Malawi, Nigeria, and Zimbabwe
Source: J Acquir Immune Defic Syndr. 2017 May 15;75(2):S233–9. doi: 10.1097/QAI.0000000000001364 (PMC5432093; doi:10.1097/QAI.0000000000001364)

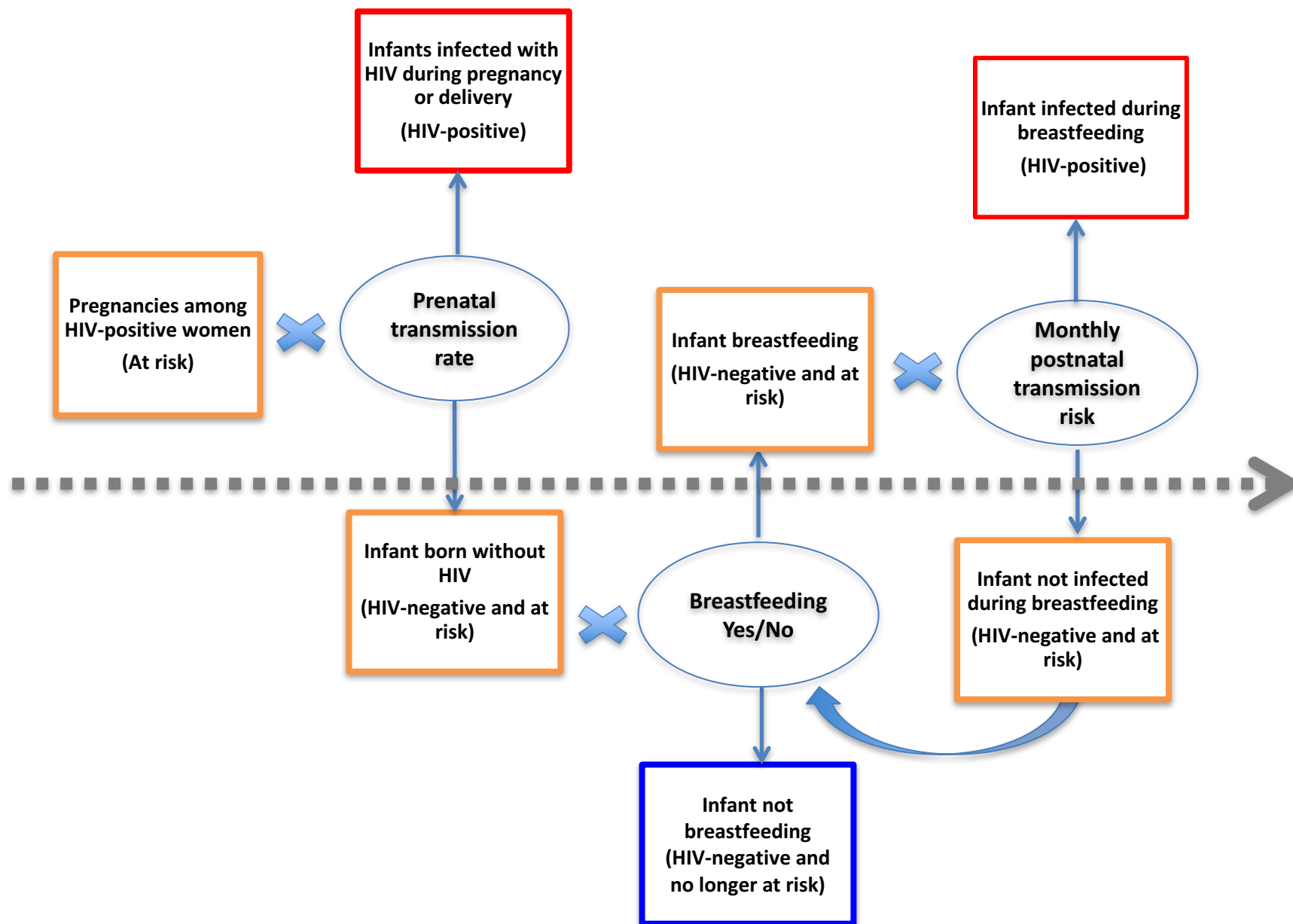

Supplement: SUPPLEMENTARY MATERIAL [file qai-75-s233-s001.pdf]
